# Urethral Stricture Disease: Challenges and Ongoing Controversies

**DOI:** 10.1155/2016/1238369

**Published:** 2016-03-14

**Authors:** Miroslav L. Djordjevic, Francisco E. Martins, Vladimir Kojovic, Dmitry Kurbatov

**Affiliations:** ^1^School of Medicine, University of Belgrade, 11000 Belgrade, Serbia; ^2^University of Lisbon, 1649-004 Lisbon, Portugal; ^3^Endocrinology Research Center, Andrology and Urology Department, Moscow 117036, Russia

Management of urethral stricture disease presents constant challenge for all reconstructive urologists. Urethral stricture disease is generally defined as stenoses that are typically long, involving broad areas of varying spongiofibrosis, and result from inflammation and/or infection, rather than trauma. Although the management of urethral strictures may be complex and challenging, very often they are treated by health care personnel without the necessary and proper training and knowledge of the current, modern, validated techniques and procedures. Notable changes in surgical approach have been adopted worldwide, resulting in significant improvement of successful outcomes and simultaneously decreasing the complication rate. Nowadays, most urethral strictures can be reconstructed in a one-stage procedure, leaving some complex cases for a less convenient, but safer, two-stage repair strategy. The exciting and enjoyable “nature” of reconstructive surgery, in general, and urethral reconstruction, in particular, is the unexpected and unpredictable nature of the stricture and, consequently, the need for the creative combination of different techniques and strategies, often involving tissue transfer procedures, either as grafts or as flaps, for achieving a successful outcome. This special issue contains a number of articles with description of different aspects, presentations, and treatments of urethral stricture disease with the aim to make further improvement of understanding and managing this severe surgical condition.

Multi-institutional review article from Portugal, India, and USA presents modality of challenging treatment of long-segment and panurethral stricture disease. Francisco E. Martins and colleagues evaluated etiology, pathogenesis, and diagnostic work-up and, finally, presented different surgical options for treatment, together with outcomes and complications. They concluded that one-stage repair with buccal mucosa grafts presents an excellent option in the treatment of long urethral stricture. However, for obliterative disease, two-stage urethroplasty offers a viable alternative.

J. Gelman and E. S. Wisenbaugh presented a review article about management of patients who suffer pelvic fracture urethral injuries which usually develop into obliterative strictures with distraction defect. They comprehensively evaluated initial management, preoperative planning, and techniques for posterior urethral stricture disease. The authors emphasize the importance of adequate vascularization of urethra for successful repair. They believe that possible future modification of operative technique could be a bulbar artery sparing surgery during posterior urethral reconstruction. Results from referral centers confirm that when open repair fails, excision and primary anastomosis still remains the procedure of choice and offers a very high success rate. In another article entitled “The Use of Flaps and Grafts in the Treatment of Urethral Stricture Disease,” the same authors described the use of versatile flaps and grafts in the various clinical presentations of anterior urethral stricture disease. Selecting the appropriate technique for each patient is highly individualized and dependent on stricture characteristics. However, the proper selection of tissue transfer technique is paramount to success. The authors provided a logical, easily comprehensible approach to the appropriate selection of grafts and flaps in urethral reconstruction, followed by practical clinical guidelines.

Another article, trying to give answers when to choose dorsal, ventral, or lateral onlay approach for buccal mucosa graft urethral reconstruction, is presented by K. Venkatesan and colleagues. The authors concluded that comparative studies are limited and choice of techniques is typically determined on location and length of stricture and surgeon preference.

The article titled “Bipolar Transurethral Incision of Bladder Neck Stenoses with Mitomycin C Injection,” written by T. D. Lyon and colleagues from Pittsburgh, presented efficacy of bipolar transurethral incision with mitomycin C injection on thirteen patients who had refractory bladder neck stenosis. Overall success was achieved in 77% (10/13) of patients. Bipolar transurethral incision with mitomycin C injection was comparable in efficacy to previously reported techniques and did not result in any serious adverse events.

Urethral stricture disease is an underrecognized and poorly reported complication after radiation therapy, and that can cause severe morbidity for cancer survivors. Radiated urethral tissue in particular poses a great challenge for the reconstructive urologist. I. Khourdaji and colleagues provided a comprehensive discussion of etiology, incidence, and available treatment options for urethral stricture disease following pelvic radiation in the article titled “Treatment of Urethral Strictures from Irradiation and Other Nonsurgical Forms of Pelvic Cancer Treatment.”

H. Okafor and D. Nikolavsky examined the impact of short-stay urethroplasty on health-related quality of life and patient's perception of timing of discharge. Over a 2-year period, a validated health-related quality-of-life questionnaire, EuroQol (EQ-5D), and additional question assessing timing of discharge were administered to all patients after urethroplasty. Postoperatively, patients were offered to be sent home immediately or to stay overnight. In this research article, the authors concluded that the majority of patients discharged soon after their procedure felt that discharge timing was appropriate and their health-related quality of life was only minimally affected.

A clinical study, published by W. Al Taweel and R. Seyam, has a goal to determine the long-term stricture-free rate after visual internal urethrotomy following initial and follow-up urethrotomies. During a period of eight years, 301 patients underwent visual internal urethrotomy. The overall stricture-free rate at the 36-month follow-up was 8.3% with a median time to recurrence of 10 months. The authors confirmed that visual internal urethrotomy for adult male urethral stricture has poor long-term results without significant difference in the stricture-free rate between single and multiple procedures.

In a multicentric clinical study that has been conducted in Italy and two centers from Belgium, M. Beysens and colleagues evaluated alterations in sexual function and genital sensitivity after anastomotic repair and free graft urethroplasty for bulbar urethral strictures. The patients who underwent anastomotic repair or free graft urethroplasty were prospectively evaluated before urethroplasty and 6 weeks and 6 months after urethroplasty. Evaluation included standardized questionnaires as IPSS, IIIEF-5, and Ejaculation/Orgasm Score and questions on genital sensitivity. The authors concluded that anastomotic repair is associated with a transient decline in erectile and ejaculatory function, and that was not observed with free graft urethroplasty. Bulbar anastomotic repair and free graft urethroplasty are likely to alter genital sensitivity. However, it should be noted that the authors are highly experienced and expert urologists, and results from any surgery performed at center of excellence may not be generalizable.

Finally, the management of urethral stricture disease is continually evolving. Although numerous strategies are available, there is still no single optimum solution suitable for all conditions. The clinical selection of stricture recurrence prevention techniques should be carefully tailored to every individual patient. Last but not least, reconstructive urologist must be familiar with a variety of these techniques, to ensure the use of the best one, as dictated by situation.

*Miroslav L. Djordjevic* *Francisco E. Martins* *Vladimir Kojovic* *Dmitry Kurbatov*

